# Regulatory network of *GATA3* in pediatric acute lymphoblastic leukemia

**DOI:** 10.18632/oncotarget.16424

**Published:** 2017-03-21

**Authors:** Qianqian Hou, Fei Liao, Shouyue Zhang, Duyu Zhang, Yan Zhang, Xueyan Zhou, Xuyang Xia, Yuanxin Ye, Hanshuo Yang, Zhaozhi Li, Leiming Wang, Xi Wang, Zhigui Ma, Yiping Zhu, Liang Ouyang, Yuelan Wang, Hui Zhang, Li Yang, Heng Xu, Yang Shu

**Affiliations:** ^1^ Department of Laboratory Medicine, Precision Medicine Center, State Key Laboratory of Biotherapy and Precision Medicine Key Laboratory of Sichuan Province, West China Hospital, Sichuan University and Collaborative Innovation Center, Chengdu, Sichuan, China; ^2^ Department of Thoracic Oncology, Cancer Center, State Key Laboratory of Biotherapy, West China Hospital, Sichuan University, Chengdu, Sichuan, China; ^3^ State Key Laboratory of Biotherapy, West China Hospital, Sichuan University and Collaborative Innovation Center, Chengdu, Sichuan, China; ^4^ Department of Molecular Biology, Baylor College of Medicine, Houston, Texas, USA; ^5^ Department of Microbiology, Immunology and Molecular Genetics, University of California at Los Angles, Los Angles, California, USA; ^6^ Department of Pediatric Hematology/Oncology, West China Second Hospital, Sichuan University, Chengdu, Sichuan, China; ^7^ Department of Pharmaceutical Sciences, St. Jude Children's Research Hospital, Memphis, TN, USA; ^8^ Department of Laboratory Medicine, Research Center of Clinical Laboratory Medicine, West China Hospital, Sichuan University, Chengdu, Sichuan, China

**Keywords:** GATA3, acute lymphoblastic leukemia, tissue-specific regulation network, microarray datasets

## Abstract

*GATA3* polymorphisms were reported to be significantly associated with susceptibility of pediatric B-lineage acute lymphoblastic leukemia (ALL), by impacting on *GATA3* expression. We noticed that ALL-related *GATA3* polymorphism located around in the tissue-specific enhancer, and significantly associated with GATA3 expression. Although the regulatory network of *GATA3* has been well reported in T cells, the functional status of *GATA3* is poorly understood in B-ALL. We thus conducted genome-wide gene expression association analyses to reveal expression associated genes and pathways in nine independent B-ALL patient cohorts. In B-ALL patients, 173 candidates were identified to be significantly associated with *GATA3* expression, including some reported *GATA3*-related genes (e.g., *ITM2A*) and well-known tumor-related genes (e.g., *STAT4*). Some of the candidates exhibit tissue-specific and subtype-specific association with *GATA3*. Through overexpression and down-regulation of *GATA3* in leukemia cell lines, several reported and novel *GATA3* regulated genes were validated. Moreover, association of *GATA3* expression and its targets can be impacted by SNPs (e.g., rs4894953), which locate in the potential GATA3 binding motif. Our findings suggest that *GATA3* may be involved in multiple tumor-related pathways (e.g., STAT/JAK pathway) in B-ALL to impact leukemogenesis through epigenetic regulation.

## INTRODUCTION

Acute lymphoblastic leukemia (ALL) is one of the most common pediatric cancers [1], and leukemogenesis has been considered to be impacted by both environmental and genetic factors [2]. Through a series of independent genome-wide association studies (GWAS) in ethnic diverse populations, several risk loci for ALL susceptibility have been identified (e.g., *ARID5B*, *IKZF1*, *CEBPE*, *PIP4K2A*, *CDKN2A*, *GATA3*) [3–10], and validated by subsequent replication studies [11–14]. However, most of these GWAS signals are located in non-coding region of the related genes, except *CDKN2A* [9]. Nevertheless, some ALL-related single nucleotide polymorphisms (SNPs) are noted to be located in the regulatory region, and impact on gene expression (e.g., SNPs of *PIP4K2A*, and *GATA3* loci [5, 8, 15]), indicating their possible epigenetic regulation. Notably, ALL-related *GATA3* SNPs (e.g., rs3824662, located in intron3) locate in its enhancer region, with higher *GATA3* expressed in risk allele carriers of EBV virus transformed lymphoblastoid cell lines (LCL), which suggests their causal mechanisms in leukemogenesis [8]. Moreover, *GATA3* SNPs are associated with ALL susceptibility with varied odds radio (OR) in terms of different clinical characteristics, and mostly impacted by subtypes (i.e., Ph-like B cell lineage ALL) [8, 10], indicating the specific role of GATA3 in different cell type.

As a well-known transcription factor, GATA3 can bind to specific motif (e.g., consensus DNA sequence WGATAR, W = A/T and R =A/G), and is capable to function in determination of cell identity of hematopoietic system, mammary gland, and etc [16, 17], especially emerging as a critical regulator of both innate and adaptive immunity. *GATA3* expression is associated with cell-type specification, and plays an important role on the development and functions of multiple immune cell types, including T cells and B cells [17, 18]. Actually, function of *GATA3* has been firstly characterized in T cell, and is essential for Th1-Th2 commitment with higher expression level in Th2 cells [19], as a transcriptional regulator through direct action at many critical factors (e.g., cytokines, signaling molecules) [18]. Also, *GATA3* plays an important role on T cells maintenance, and is required for distinct aspects of T cell activation and proliferation in cell type-specific manner [17]. Through large efforts with experimental analyses, multiple upstream regulators and downstream targets of *GATA3* have been characterized in T cells [20]. For instance, interleukin 4 can promote *GATA3* expression through STAT6 signal [17, 19]. Also, *GATA3* is involved in multiple pathways independent of IL4-STAT6 signaling, including Notch and Wnt pathways [21–23], which are essential for T cell development.

Moreover, Knocking-out of *Gata3* in mouse results in embryonic lethal between E11 and E12, displaying massive internal bleeding, gross aberrations in fetal liver hematopoiesis, and etc [24]. Importantly, aberrant *GATA3* expression or mutations can impact on its downstream genes, thus induce dysfunctions including tumorigenesis, such as breast cancer [25, 26]. For instance, loss of *Gata3* in adult mice leads to an expansion of undifferentiated luminal cells and basement-membrane detachment, which may promote tumor dissemination [27], while rescue of *Gata3* expression reduces both tumorigenicity and metastatic potential of breast cancer cells [28, 29]. In human cancers, frequent loss-of-function of *GATA3* alteration and copy number deletions were observed in breast cancer and T cell leukemia/lymphoma recently [25, 30].

Recent studies indicate that *GATA3* can actively suppress B cells development [17, 31, 32], and deficiency of this gene results in development failure of T cells but not B cells in conditional hematopoietic knockout mouse model [33, 34], raising the possibility that *GATA3* was involved in cell-type specific regulatory network. However, despite of studies on association of *GATA3* SNPs with B-ALL susceptibility, function of *GATA3* in leukemogenesis for B lineage cells was poorly understood. It will be time and effect consuming to figure out the *GATA3*-involved regulatory network in B lineage ALL (B-ALL) with the traditional methods, especially for those unreported genes. Fortunately, array based characterization of transcriptional profiles have been conducted in multiple independent B-ALL patient cohorts. With the public resource, we conducted transcriptional wide screening in this study to effectively find the genes those are significantly related to *GATA3* expression, and built the regulatory network. Subsequent validations were also carried out for some of the candidates in ALL cell lines to evaluate the reliability of this procedure.

## RESULTS

### The top GWAS SNP for ALL susceptibility is located in the enhancer region of *GATA3* in a tissue-type specific manner

The function of *GATA3* has been largely revealed as a transcription factor and highly expressed in multiple tissues including breast, bladder, blood, skins (Supplementary Figure 1). Significant expression changes between tumor and control normal tissues were also observed in multiple types of cancers according to the dataset of The Cancer Genome Atlas (TCGA) (Supplementary Figure 2). However, opposite directions were also noticed with higher expression level in tumors (e.g., bladder cancer, cervical squamous cell carcinoma) or in normal tissues (e.g., kidney cancer) (Supplementary Figure 2), indicating the important and heterogeneity role of *GATA3* in tumorigenesis for different types of cancer. Therefore, it is important to find the regulatory network of *GATA3* in each type of cancer separately, including B-ALL.

**Figure 1:**
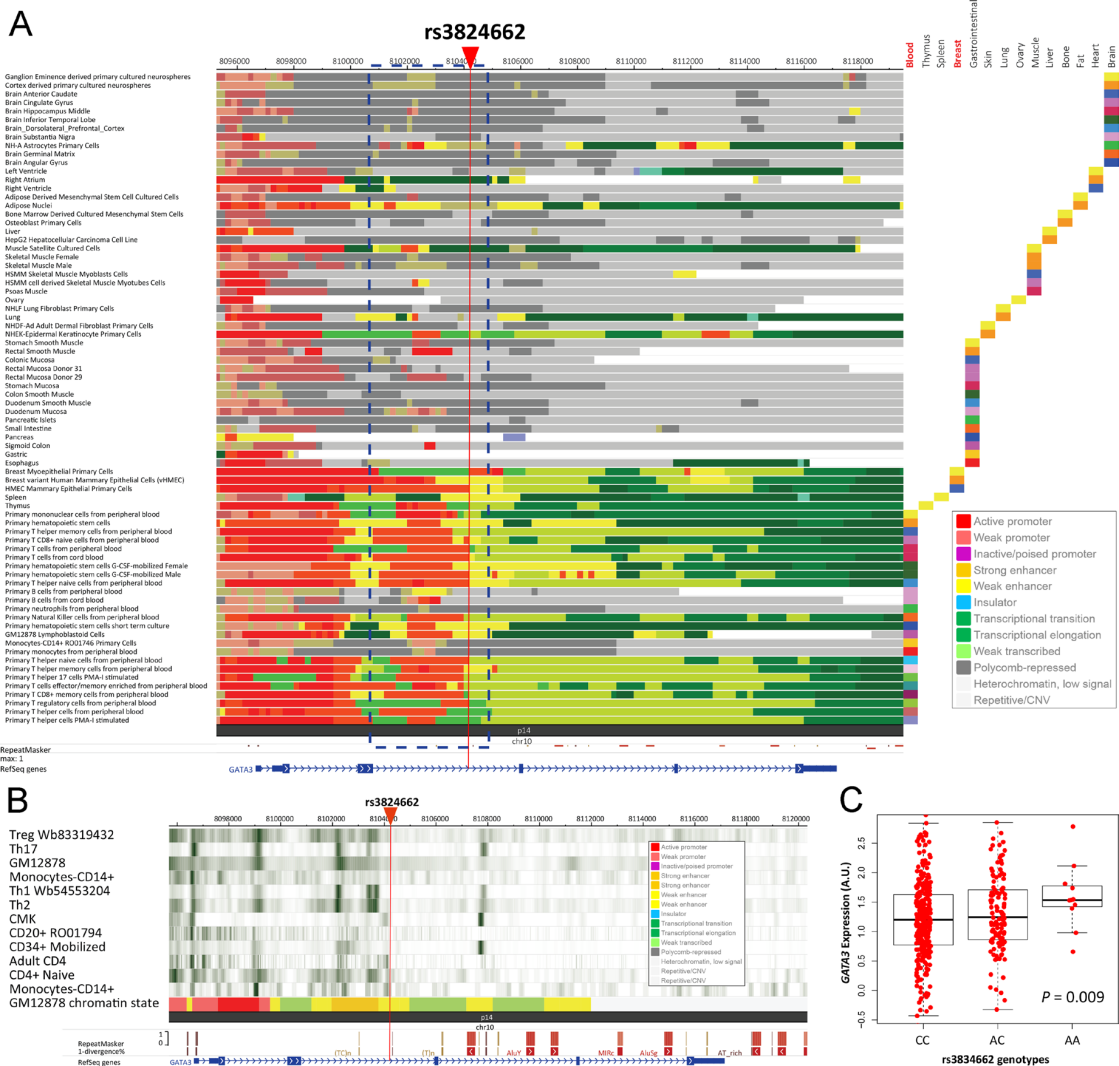
Epigenetic regulation of *GATA3* (**A**) Epigenetic elements around the top GWAS *GATA3* SNP (i.e., rs3824662) in different tissue types. Different epigenetic elements were labeled as different colors as annotation indicated, and the tissue type information was listed on the right with “Blood” and “Breast” highlighted (**B**) DNase hypersensitivity signals around rs3824662 in different types of blood cells. Strength of the binding for each transcriptional factor was illustrated according to the (**C**) Genotype-expression association between rs3824662 and *GATA3* expression in LCLs, *P* = 0.009.

Because the top SNP (i.e., rs3824662) for ALL susceptibility in *GATA3* is located in its intron region, epigenetic signals were thus analyzed with the public resource (e.g., ENCODE and ROADMAP database). Interestingly, a strong enhancer close to rs3824662 was observed in a tissue-type specific manner, and blood and breast exhibit strong signals (Figure 1A), which is consistent with their higher expression level among different tissue types (Supplementary Figure 1). Additionally, differences were also observed among hematopoietic cell types. For instance, CD34 positive cells have relatively weaker DNAase hypersensitivity signal around rs3824662 compared to other type of hematopoietic cells, indicating the varied role of rs3824662 on *GATA3* regulation in different development stage of hematopoietic cells (Figure 1B). Additionally, risk allele of rs3824662 is significantly related to higher expression level of *GATA3* in LCLs from diverse ethnicities, (*P* = 0.009 after adjust for ethnicity) (Figure 1C), indicating overexpression of *GATA3* may increase the risk of leukemogenesis through SNP-induced epigenetic regulation.

### Multiple genes are significantly associated with *GATA3* expression in B-ALL

**Figure 2:**
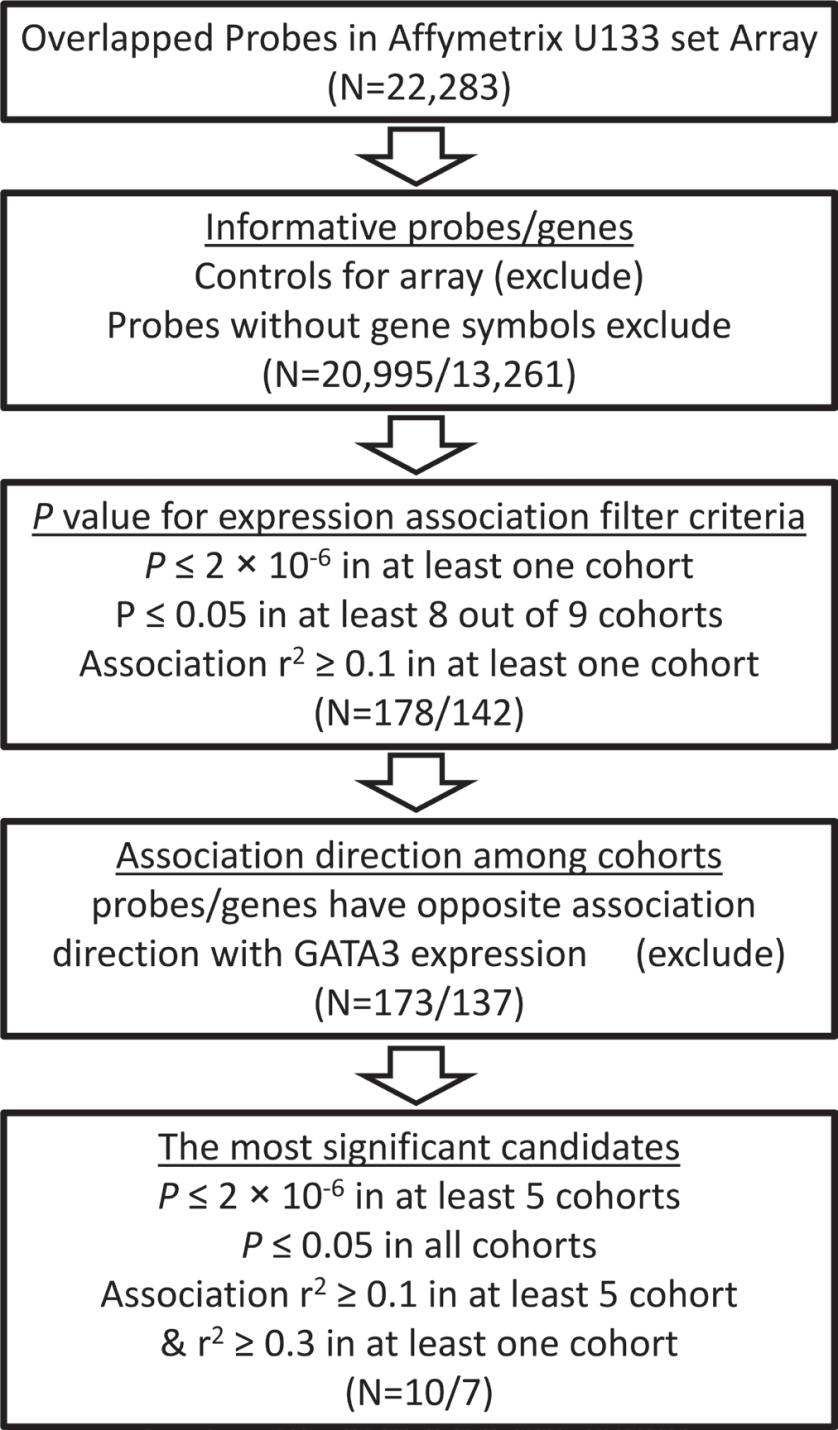
Flow chart for *GATA3*-related genes screening pipeline

**Figure 3:**
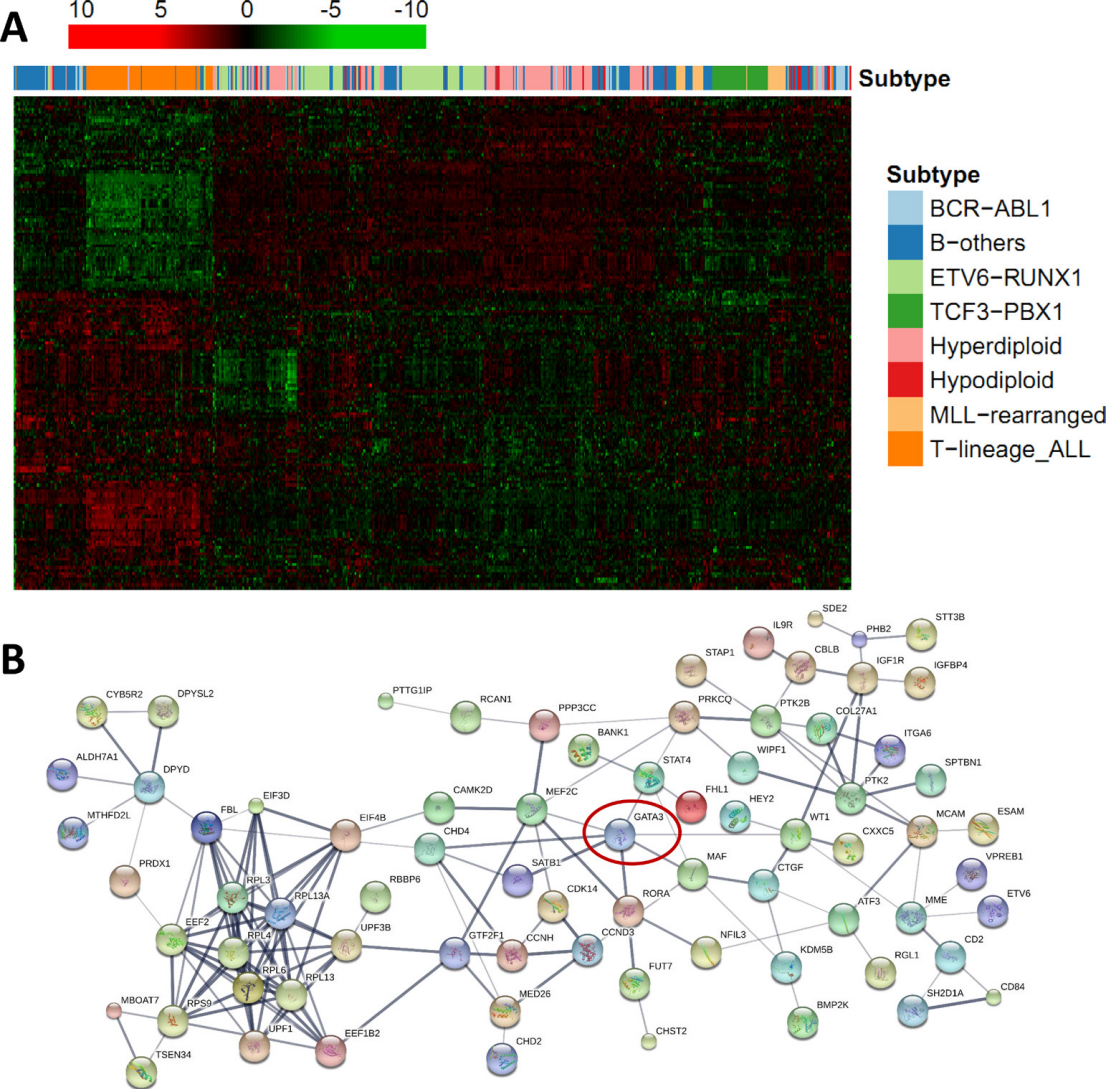
Regulatory statement of *GATA3* and its related candidates in B-ALL (**A**) Expression clustering illustration of *GATA3*-related 137 candidates, with B-ALL subtypes was labeled above with different colors indicated. (**B**) Protein-protein interaction network of *GATA3*-related genes. Line thickness indicates the strength of data support, and nodes that disconnected with the main network were hide.

Expression array data from nine independent ALL patient cohorts were downloaded from the public resource (Table 1). Association of *GATA3* expression with all the rest genes were estimated by using linear regression model. To find the potential expression related genes and build the co-expression network of *GATA3* in B-ALL, a series of filter steps were applied for candidate selection, including strict *P value* cutoff, *r*^2^, and consistent direction for association coefficient (Figure 2). Interestingly, only 5 out of 142 genes (or 5 out of 178 array probes) were filtered out because of the inconsistency direction among cohorts, indirectly proving the high reliability of the selected candidates. Totally 83 and 54 genes were positively and negatively related to *GATA3* expression, respectively (Supplementary Table 1). Due to the large sample size and availability of clinical information, data from GSE33315 was used for further analyses (with 173 probes for 137 genes have available expression information). Expression level of *GATA3* in B-ALL is significantly higher than that in CD19 positive cells, and similar as that in *CD34* positive cells from healthy people (Supplementary Figure 3). The highest *GATA3* expression was observed in B-others subtype, possibly because Ph-like ALL was included in such subtype. Interestingly, these *GATA3*-related genes are tend to be clustered in ALL subtypes in heatmap, indicating their different roles on leukemia subtypes (Figure 3A). Among these candidates, some genes have already been reported as upstream regulators (e.g., *SATB1* [21]) or downstream targets (e.g., *ITM2A* [20, 35]) in T cells (Supplementary Table 1), exhibiting the ubiquitous *GATA3*-related network in different cell types as well as the reliability of our screening procedure. The candidates was also listed, which are significantly related to *GATA3* expression in all patient cohorts with *P* ≤ 2 × 10^−6^ and *r*^2^ ≥ 0.1 in at least 5 cohorts (Table 2). Interestingly, *STAT4*, which is involved in JAK/STAT pathway, has been found as one of the strongest candidates. Considering that *GATA3* SNP is more related to Ph-like ALL, which is enriched in JAK pathway alteration, *GATA3* may be involved in B-ALL leukemogenesis through inducing *STAT4* overexpression and activating the JAK/STAT pathway. Additional, we also found another novel target (i.e., *ETV6*), alteration of which is frequently observed in leukemia in germline [36] or somatic level. Next, we conducted pathway analyses by using online tools (e.g., DAVID Functional Annotation Tools), and found that two gene sets were significantly enriched in *GATA3*-related genes (i.e., “Cyclin” and “RNA polymerase II regulatory region sequence specific DNA binding “ Supplementary Table 2), suggesting *GATA3* may impact cell cycle and involved in complicated transcriptional regulation to induce leukemogenesis. Additionally, protein-protein interaction network of these candidates was also illustrated with STRING, IntAct and BioGRID to indicate the known interactions (Figure 3B), those genes that were not illustrated may be considered as novel members in *GATA3* regulatory network specific in B-ALL.

**Table 1:** Summary information for the B-ALL microarray datasets

Year	Author[*]	Dataset ID	Age group	Analyses
2008	Bhojwani D et al. [44]	GSE7440	pediatric	
2010	Kang H et al. [45]	GSE11877	pediatric	
2009	Bungaro S et al. [46]	GSE10792	pediatric	
2009	den Boer ML et al. [47]	GSE13351	pediatric	Discovery
2009	den Boer ML et al. [47]	GSE13425	pediatric	
2004	Holleman A et al. [48]	GSE635	pediatric	
2008	Sorich MJ et al. [49]	GSE10255	pediatric	
2006	Kirschner-Schwabe R et al. [50]	GSE4698	pediatric	
2012	Zhang J et al. [51]	GSE33315	pediatric	
2009	Haferlach T et al. [52]	GSE13204	all ages	Validation

* number in the brackets represent the references in the manuscript.

**Table 2:** Strongest GATA3-related candidates in different datasets

Gene	Probe ID	Value index	GSE10255	GSE10792	GSE11877	GSE13351	GSE13425	GSE33315	GSE4698	GSE635	GSE7440
*NPY*	206001_at	*P* value	8.9 × 10^−16^	0.003	2.8 × 10^−7^	9.2 × 10^−5^	2.5 × 10^−10^	2.3 × 10^−8^	0.002	2.1 × 10^−28^	0.016
		coeff	−0.43	−0.29	−0.26	−0.36	−0.48	−0.19	−0.31	−0.58	−0.21
		*r*^2^	0.33	0.1	0.12	0.15	0.23	0.06	0.14	0.51	0.05
*LGMN*	201212_at	*P* value	4 × 10^−8^	0.003	0.002	5.1 × 10^−8^	1.2 × 10^−7^	7.5 × 10^−8^	0.002	4.5 × 10^−16^	0.004
		coeff	−0.33	−0.46	−0.18	−0.56	−0.37	−0.16	−0.58	−0.52	−0.24
		*r*^2^	0.17	0.09	0.04	0.27	0.16	0.06	0.14	0.32	0.07
*WT1*	206067_s_at	*P* value	6.9 × 10^−6^	0.001	6.1 × 10^−8^	1.3 × 10^−11^	4.4 × 10^−9^	2.2 × 10^−11^	0.01	3.1 × 10^−5^	2.6 × 10^−7^
		coeff	0.27	0.32	0.3	0.54	0.42	0.2	0.4	0.3	0.33
		*r*^2^	0.11	0.11	0.13	0.39	0.2	0.09	0.09	0.09	0.23
*MAST4*	222348_at	*P* value	5.0 × 10^−11^	7.7 × 10^−9^	2.6 × 10^−7^	1.9 × 10^−9^	0.002	1.7 × 10^−17^	8.1× 10^−6^	0.004	4.9 × 10^−6^
		coeff	0.59	2.09	0.29	0.74	0.34	0.38	2.84	0.28	0.4
		*r*^2^	0.23	0.34	0.12	0.32	0.05	0.14	0.28	0.04	0.19
*MAST4*	210958_s_at	*P* value	4.2 × 10^−19^	1.4 × 10^−7^	1.6 × 10^−5^	3.5 × 10^−6^	0.032	3.4 × 10^−25^	0.001	2.0 × 10^−8^	2.1 × 10^−7^
		coeff	0.84	1.92	0.33	0.76	0.3	0.5	2.73	0.69	0.55
		*r*^2^	0.39	0.29	0.08	0.21	0.02	0.2	0.17	0.16	0.24
*MAST4*	40016_g_at	*P* value	1.7 × 10^−23^	1.3 × 10^−10^	2.4 × 10^−14^	6.1 × 10^−11^	3.0 × 10^−6^	1.1 × 10^−29^	3.9 × 10^−5^	2.6 × 10^−14^	5.6 × 10^−9^
		coeff	0.82	1.4	0.57	0.71	0.5	0.5	1.7	0.7	0.45
		*r*^2^	0.46	0.4	0.24	0.37	0.13	0.23	0.24	0.28	0.29
*FBL*	211623_s_at	*P* value	6.0 × 10^−10^	0.003	2.8 × 10^−5^	5.2 × 10^−9^	9.2 × 10^−10^	1.5 × 10^−8^	0.031	3.4× 10^−18^	0.001
		coeff	1.22	1.25	0.59	1.71	1.22	0.5	0.67	1.66	0.63
		*r*^2^	0.21	0.1	0.08	0.31	0.21	0.06	0.06	0.35	0.11
*CD84*	205988_at	*P* value	1.4 × 10^−16^	3.4 × 10^−8^	0.007	3.8 × 10^−7^	0.001	7.6 × 10^−23^	0.025	2.9 × 10^−8^	0.003
		coeff	1.13	1.28	0.24	0.89	0.58	0.67	1.25	0.85	0.43
		*r*^2^	0.35	0.31	0.03	0.24	0.07	0.18	0.07	0.16	0.08
*ITM2A*	202747_s_at	*P* value	2.6 × 10^−39^	3.1 × 10^−16^	5.2 × 10^−25^	8.7 × 10^−14^	5.0 × 10^−8^	4.8 × 10^−51^	5.2 × 10^−6^	2.0 × 10^−22^	6.0 × 10^−15^
		coeff	0.57	0.98	0.7	0.64	0.54	0.45	0.57	0.65	0.68
		*r*^2^	0.66	0.57	0.4	0.46	0.17	0.37	0.29	0.42	0.46
*ITM2A*	202746_at	*P* value	2.6 × 10^−47^	1.1 × 10^−14^	2.3 × 10^−21^	1.9 × 10^−16^	2.3 × 10^−10^	2.5 × 10^−57^	2.5 × 10^−7^	3.7 × 10^−25^	9.8 × 10^−19^
		coeff	0.7	0.77	0.6	0.72	0.54	0.57	0.49	0.57	0.74
		*r*^2^	0.73	0.53	0.35	0.53	0.23	0.41	0.36	0.46	0.55

### *GATA3*-related genes exhibit tissue and subtype specific association

**Figure 4:**
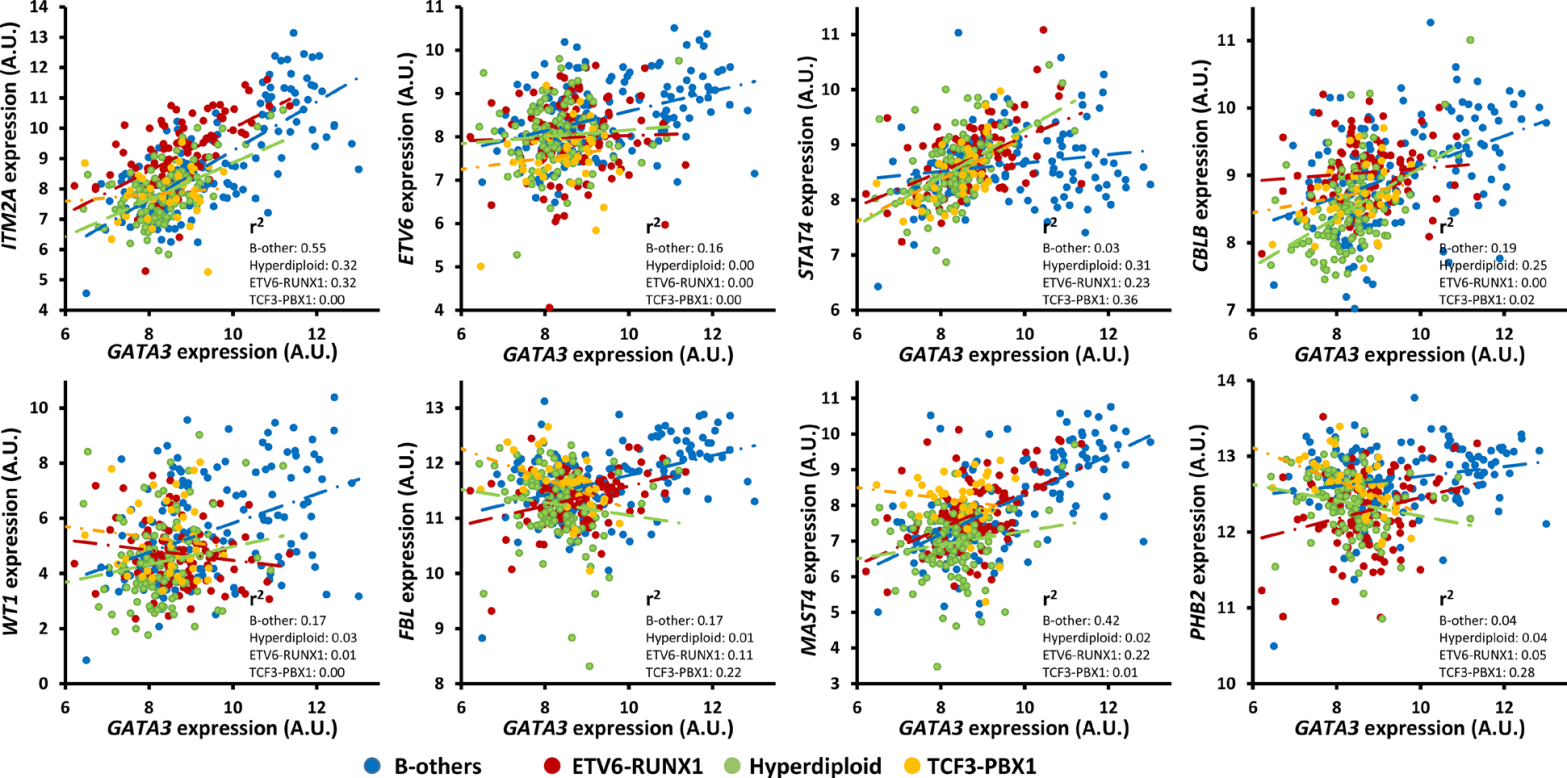
Expression association status of *GATA3* with some of the important candidates in different subtypes of B-ALL (i.e., B-others, ETV6-RUNX1, Hyperdiploid, and TCF3-PBX1) in the largest available pediatric B-ALL cohort (i.e., GSE33315) with the *P values* listed in Table 1 and Supplementary Table 3

Since the clusters of the GATA3-related gene closely match B-ALL subtypes (described above), the role of *GATA3* in different subtypes of B-ALL was checked in the largest pediatric B-ALL cohort (GSE33315) by analyzing each subtype separately (Supplementary Table 3). Most of candidates were only significant association with *GATA3* expression in some of the subtypes, partially because of the small sample size in some subtypes such as BCR-ABL and MLL rearrangement subtypes. To exclude the impact of sample size, we next analyzed the subtypes with at least 90 patients (i.e., ETV6-RUNX1, Hyperdiploid, and B-other subtype), only 36 out of 136 genes are significantly associated with *GATA3* expression in all three subtypes. All of them have the same direction except *PHB2* (Figure 4), which is positively related to *GATA3* expression in ETV6-RUNX1 and B-other subtypes but negatively related to that in hyperdiploid subtype (Figure 4). For the seven strongest candidates described above, *ITM2A*, and *MAST4* exhibit statistically significant in three subtypes with varied coefficient value, and the rest 5 genes only exhibit significance in one or two subtypes (Figure 4, and Supplementary Table 3), suggesting the different regulatory network of *GATA3* in each subtypes.

**Figure 5:**
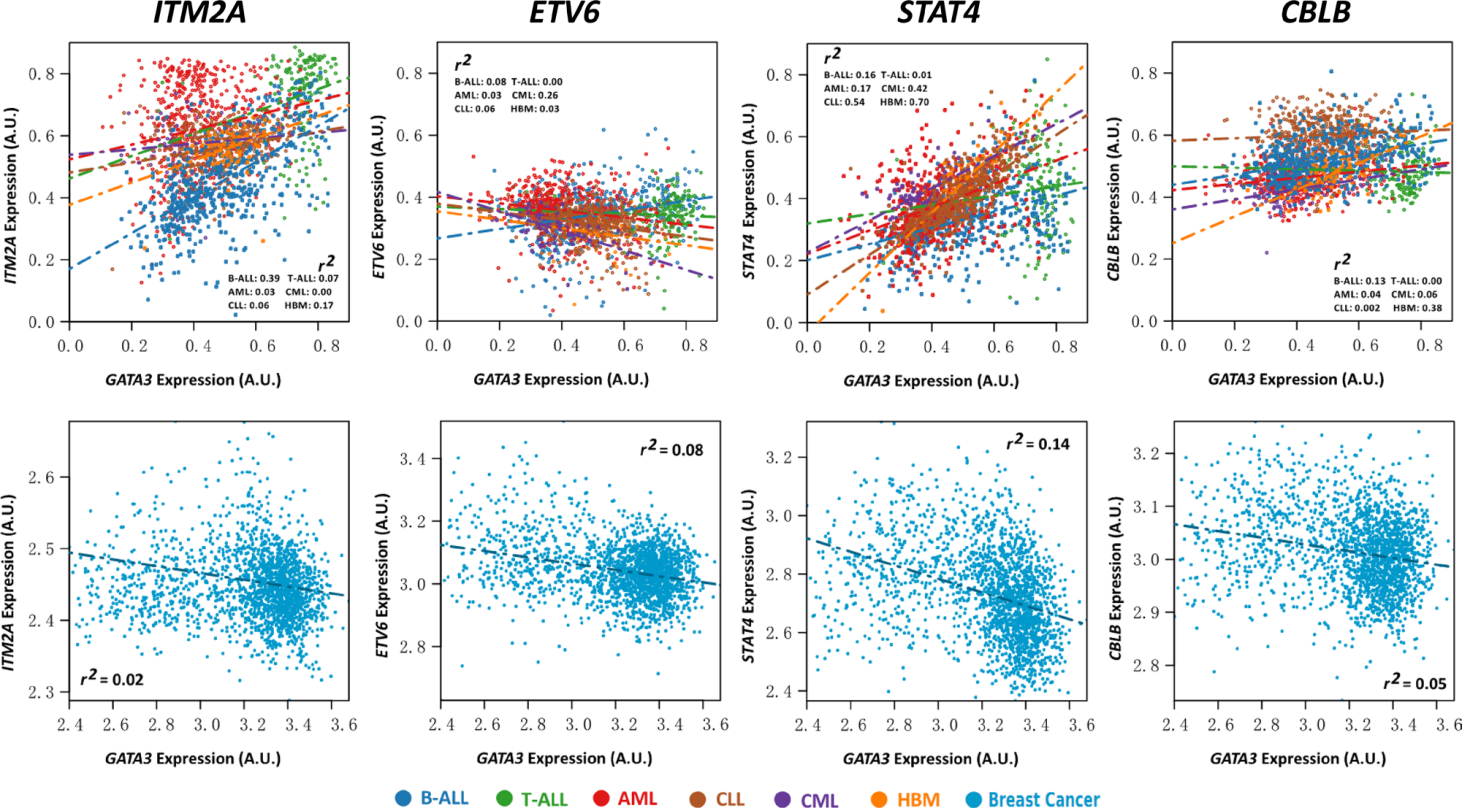
Expression association status of *GATA3* with *ITM2A, ETV6, STAT4*, and *CBLB* in different types of leukemia, including B-ALL (*N* = 576), T-ALL (*N* = 174), AML (*N* = 542), CLL (*N* = 448), CML (*N* = 76), and healthy bone marrow (HBM, *N* = 74) based on GSE13204, and breast cancer based on EGAS00000000083 (*N* = 1,992)

In another hand, we also checked the association status in a dataset containing ALL, acute myeloid leukemia (AML), chronic lymphoblastic leukemia (CLL), and chronic myeloid leukemia (CML) patients in all stage of ages at diagnosis (i.e., GSE13204). Not surprisingly, most of the candidate genes (98.5%, 135/137) reached statistical significance in B-ALL, and all of them have the same direction with the previous results. However, the consistent rate dropped to 36.5% (50/137), 75.9% (104/137), 64.9% (89/137), and 40.1% (55/137) in T-ALL (*N* = 174), CLL (*N* = 448), AML (*N* = 542), and CML (*N* = 76), respectively. Among the rest filtered genes, we noticed that 20% (10/50 in T-ALL), 25.9% (27/104 in CLL), 43.8% (39/89 in AML), and 16.3% (9/55, in CML) were even in the opposite association direction with *GATA3* to that in ALL (Supplementary Table 4). We next evaluated the candidate genes in breast cancer, on which *GATA3* also plays an important role according to the reports. Among the available gene expression information (157 genes in 1,992 patients), only 19% genes (30/157) exhibit *P* < 0.05 and *r*^2^ > 0.1. In addition, 50% (15/30) of the rested candidates have the opposite association direction with *GATA3* to that in ALL (Figure 5 and Supplementary Table 5). Taking *STAT4* as an example, which is positively related to *GATA3* expression in healthy bone marrow (*P* = 5.7 × 10^−21^, and *r*^2^ = 0.7), the association got weak gradually in CLL (*P* = 4.7 × 10^−78^, and *r*^2^ = 0.54), CML (*P* = 1.2 × 10^−10^, and *r*^2^ = 0.42), AML (*P* = 1.2 × 10^−23^, and *r*^2^ = 0.17), B-ALL (*P* = 1.7 × 10^−23^, and *r*^2^ = 0.16), and T-ALL (*P* = 0.16, and *r*^2^ = 0.005) (Supplementary Table 4), and even negatively related to *GATA3* expression in breast cancer (*P* = 1.2 × 10^−66^, and *r*^2^ = 0.14) (Supplementary Table 5). For *ETV6*, expression of this gene is positively associated with *GATA3* in B-ALL only, and with the opposite direction in all other types of leukemia, breast cancer as well as the healthy bone marrow, indicating its specific role on B-ALL leukemogenesis with *GATA3* regulation. In conclusion, there are large differences in *GATA3*-related genes and corresponding regulatory network in varied tissues and subtypes.

### Multiple leukemia or cancer related genes are associated with *GATA3* expression in cell lines

**Figure 6:**
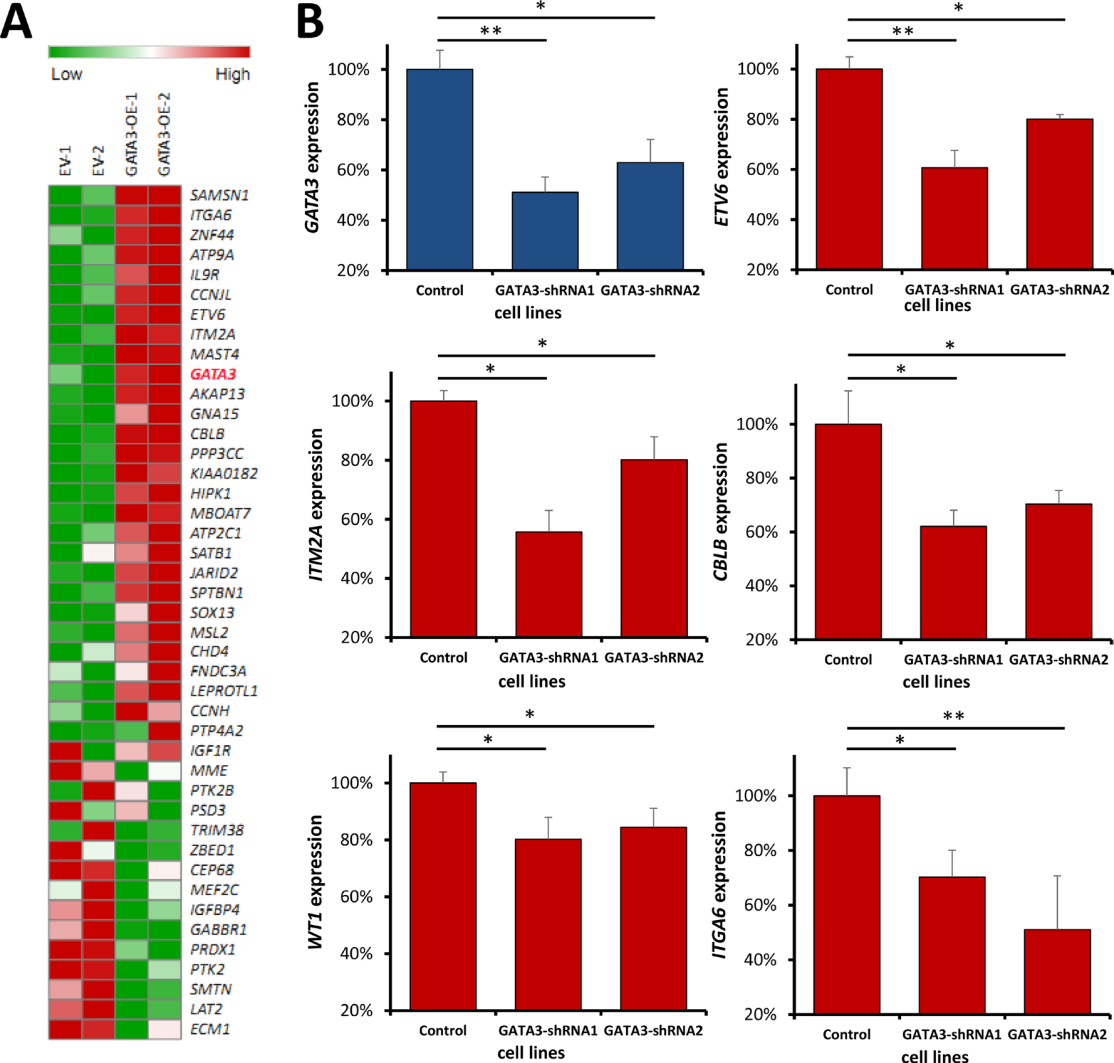
Impact of *GATA3* expression changes on the candidates in leukemia cells Expression changes of *GATA3*-related candidates in *GATA3* overexpression and *GATA3* down-regulated cells. (**A**) Heatmap for the candidate gene expression in *GATA3* overexpression cells. The most significant genes were listed on the top (positive related) and the bottom (negative related) (**B**) The expression changes of *ETV6, ITM2A, WT1, ITGA6* and *CBLB* were detected in *GATA3* down-regulated cells. (* and ** indicate *P* < 0.05 and *P* < 0.01, respectively).

Although we have found the candidates that are significantly associated with *GATA3* expression, and build regulatory network based on the known resources, it is also important to figure out the detail relationship between *GATA3* and these genes. We assumed these candidates can be upstream regulators or downstream targets of *GATA3* through direct or indirect interactions. Therefore, we retrieved the expression data of the candidates from Nalm6 cells with *GATA3* overexpression or empty vector control. Available expression information were got for 43 genes, which had present expression in control or/and *GATA3* overexpression cells. Not surprisingly, 27 out of 43 candidates were significantly changed after *GATA3* overexpression (e.g., *ETV6* and *WT1*), with the same association direction as described above (Figure 6A and Supplementary Table 6). For those were not significant changed genes, we considered them as potential upstream of *GATA3*, such as *SATB1*, which has been reported as regulator of *GATA3* in T cell. Additionally, we also picked some of the strong candidates (e.g., *ITM2A*) for analyses with shRNA system in other leukemia cell lines for validation. Cells with *GATA3* knocking down exhibited consistent changes as well (Figure 6B), indicating the reliability of our analyses.

### Loss of GATA3 binding motif induced by SNP can impact association of *CBLB* with *GATA3* expression

**Figure 7:**
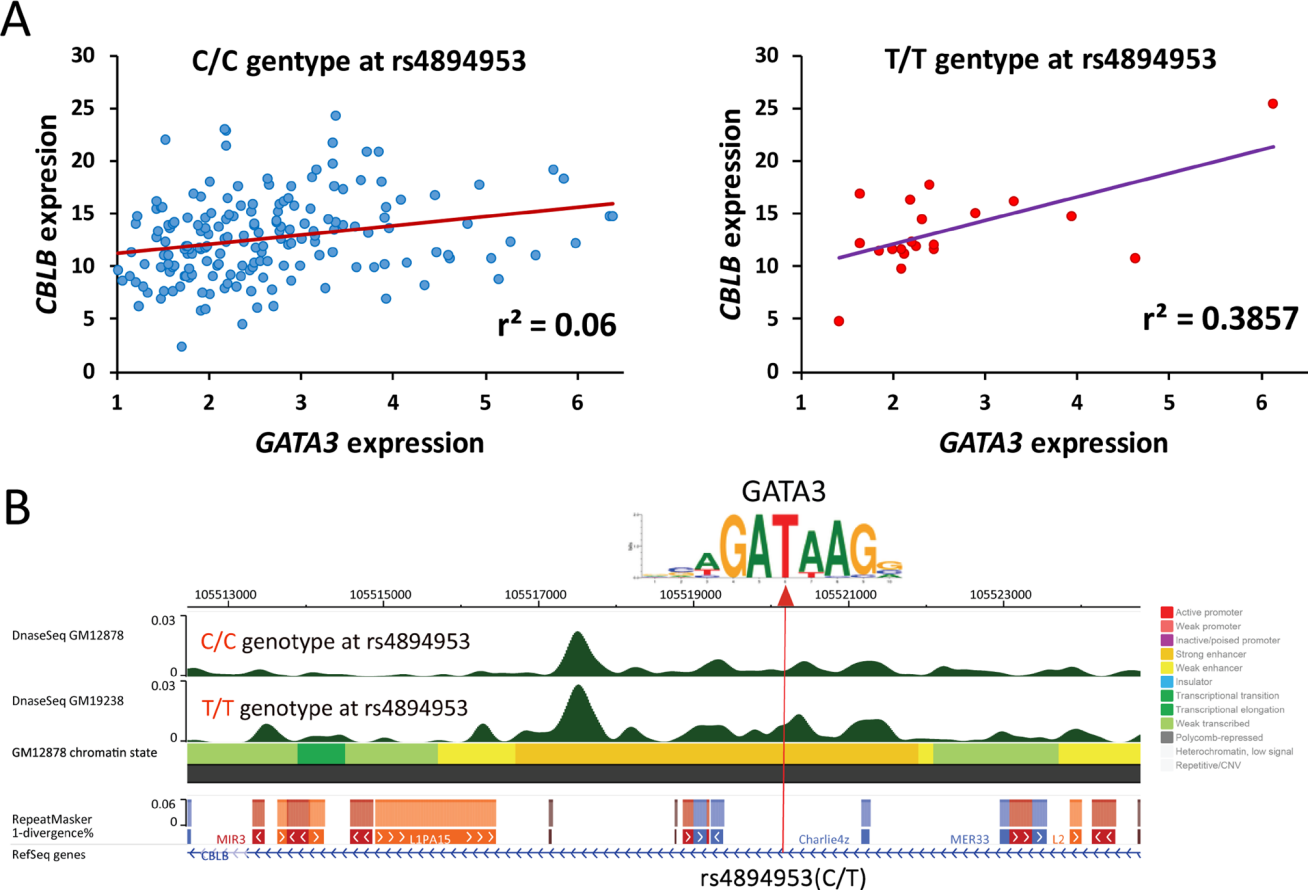
Impact of SNP genotypes on expression association between *GATA3* and its downstream target (**A**) Expression association status between *GATA3* and *CBLB* in terms of C/C and T/T genotypes at rs4894953. (**B**) Impact of different allele on GATA3 binding motif and the epigenomic signals in LCL with C/C and T/T genotypes at rs4894953.

We next checked whether the expression of the candidates can be impacted by SNPs, which alter the *GATA3* binding affinity through breaking the conserved “GATA” motif. Interestingly, *CBLB*, which is the potential downstream target of *GATA3* in leukemia as well as LCLs according to our results, contains one SNP (i.e., rs4894953) in its enhancer region. rs4894953 and its flanking nucleotide acids form a sequence of “GA(T/C)A”, in which *GATA3* is more likely to bind to this motif in individuals with T allele at this SNP. Therefore, we conducted genotype-specific expression association analyses in LCLs, which comprehensive information for both SNP genotypes and gene expression were available. We separated the individuals in terms of genotypes of rs4894953, and checked the association of *GATA3* expression with *CBLB*. Interestingly, although significant association of these two genes can be detected in both C/C (*P* = 0.0008) and T/T (*P* = 0.003) genotype groups, large difference was observed in terms of *r*^2^, (i.e., *r*^2^ = 0.06 and 0.39 in C/C and T/T genotype groups, respectively) (Figure 7A). Additionally, we also checked the available epigenetic signal in LCLs, and noticed that the DNase I hypersensitivity signal is stronger in GM19238 (T/T at rs4894953) than that in GM12878 (C/C at rs4894953) around the SNP (Figure 7B). These results indicated that the expression of *GATA3*-related candidates can be strongly impacted by SNPs those locate in “GATA” motif, and further suggested the reliability of the candidates we screened out.

## DISCUSSION

Due to the varied roles of *GATA3* on different tissue types, it is important but also time/effect-consuming to find the regulatory network of *GATA3* in each type of cancer separately. We assumed that the genes involved in the same regulatory network will be related in terms of expression level among patients, and the transcription factor and its direct target will exhibit the most significant association. Therefore, it will be easy and effective to screen the *GATA3*-related genes through whole transcriptome-wide association by using the public available microarray datasets. Interestingly, multiple GWASs revealed strong association of *GATA3* SNP with ALL susceptibility, especially in Ph-like subtypes, and the risk allele of the top GWAS SNP is related to higher expression of *GATA3*. Therefore, the mechanism of how *GATA3* involved in B lineage leukemogenesis can be studied on its upstream and downstream signals in leukemia cells from B-ALL patients. Finally, we found 137 genes that are potential involved in *GATA3*-related regulatory network with nine independent pediatric ALL patient cohorts, and got validated in another leukemia cohort containing all ages of B-ALL patients. Interestingly, all of strongest candidates are significantly associated with *GATA3* expression in B others, which could reflect the risk allele enrichment of *GATA3* SNP in Ph-like ALL subtype. However, due to the different association status of *GATA3* with its related gene in different subtypes of B-ALL, analyses within a certain subtype should be done if the information can be got from more larger size of patient cohorts. Notably, some of the candidates have been reported to be upstream regulator (e.g., *SATB1* [21]) or downstream targets (e.g., *ITM2A* [20, 35]) of *GATA3* in other types of cells, indicating the reliability of our methods. Besides, our results provided a clue for further studies on how *GATA3* acted in leukemogenesis.

Interestingly, some of the well-known cancer related genes were found, such as *STAT4*. Actually, STAT4 is involved in JAK/STAT pathway, and constitutive activation of JAK-STAT was recognized as being associated with malignancy, including leukemia [37]. Moreover, *GATA3* has stronger effects in Ph-like ALL, in which gain-of-function of JAK mutations is enriched, raising the possibility that *GATA3* increases the leukemogenesis risk through activating JAK-STAT signaling. Consistently, *STAT4* expression decreases in leukemia cells treated with shRNA against *GATA3*, which was also confirmed in previous reports [38]. Paradoxically, *STAT4* is negatively related to *GATA3* expression in breast cancer, probably because of the tissue specific role of *GATA3*. Actually, we noticed that lots of *GATA3*-related genes in B-ALL lost their association with *GATA3* expression in breast cancer, or even exhibit the opposite association directions (e.g., *STAT4*), providing the possible explanation of the opposite role of *GATA3* in different cancer types (e.g., potential tumor suppressor in breast cancer but oncogene in leukemia). Not surprisingly, similarity of association status and regulatory network may increase in more related cell types. For instance, number of *GATA3*-related genes, which exhibit same significant association direction with that in pediatric B-ALL, is the largest in all ages of B-ALL, and gradually decreases in B lineage chronic lymphocytic leukemia, myeloid leukemia, and breast cancer.

As described above, the candidates can be upstream regulator or downstream targets of *GATA3* with direct or indirect binding. In T cells, *ITM2A* is a direct target of *GATA3* [35], *ITGA6* can be indirectly down regulated by *GATA3* via microRNA-29b [39], whereas *SATB1* acts as upstream regulator and positively regulates *GATA3*. Therefore, knocking-down *GATA3* can largely reduce expression of *ITM2A*, slightly for *ITGA6* but not for *SATB1*, which can be validated in cellular experiments, indicating the ubiquitous association of some candidates among different cell types. Moreover, candidates can be impacted by SNPs in “GATA” motif in their regulatory elements, as direct downstream targets of *GATA3*, such as rs4894953, located in the enhancer region of *CBLB*, appears in the C allele, then expression of *CBLB* is down-regulated compared with T allele as the result of losing GATA3 binding site characterized in *CBLB*. Some of the other candidates have been linked to *GATA3* through PPI prediction, and the validations for well-known cancer related genes should be first priority to reveal the mechanism of *GATA3* induced leukemogenesis. In another hand, risk allele *GATA3* SNPs are associated with higher risk of B-ALL relapse as well, suggesting higher expression of *GATA3* will result in poor treatment outcomes. Recently, GATA3 overexpression has been reported to be associated with poor overall survival in Peripheral T-cell lymphoma [40], but a favorite prognostic factor for breast cancer. We assumed that the paradox might be explained by the *GATA3* related candidates with opposite directions.

Importantly, pipeline we developed can be expanded to screen the regulatory network of other important genes in different cancer types, especially for those transcription factors. In this study, we used a very strict criteria to screen the strongest candidates, which can induce high rate of false negative. When this pipeline will be used in other studies, multiple factors should be considered to balance the false negative and false positive, including sample size, number of available cohorts, heterogeneity of the patients, and etc. Moreover, experimental validations are always needed for the final determination. Notably, this method can't be used to find out the gene-related candidates through other mechanisms, such as protein-protein interaction or post-transcriptional/post-translational modifications.

In conclusion, we have used a series of public available microarray datasets, and developed an effective pipeline to find 173 *GATA3*-related genes in B-ALL. With the bioinformatics analyses and cellular experiment validations, multiple potential *GATA3* related genes (e.g., *ETV6*) and signaling pathways (JAK/STAT and cell cycle pathways) were determined in ubiquitous or B-ALL specific manner. We conclude that risk allele of *GATA3* SNP induces overexpression of *GATA3*, and subsequently impacts on the regulatory network of *GATA3* to increase the susceptibility for B-ALL leukemogenesis.

## MATERIALS AND METHODS

### Epigenetics regulation illustration and genotype-expression association analyses

Online tools (i.e., Epigenome Browser [41]) was used to illustrate the epigenetic element around SNPs of *GATA3* and *CBLB* by introducing Roadmap and ENCODE information from multiple tissue and cell types. Expression level of *GATA3* gene was obtained from public RNA-seq data resource of Lymphoblastoid cell lines [42], and genotypes of rs3824662 was obtained from the 1000 genome project website (http://grch37.ensembl.org/index.html). As described before, Genotype-expression association was assessed through a linear regression model for the available individuals (*N* = 441) [43].

### Expression microarray datasets searching and association analyses

Expression level of all genes in B-ALL patients were obtained from Gene Expression Omnibus (GSE7440 [44], GSE11877 [45], GSE10792 [46], GSE13351 [47], GSE13425 [47], GSE635 [48], GSE10255 [49], GSE4698 [50], GSE33315 [51], and GSE13204 [52]). Association of *GATA3* expression of all the rest genes were estimated by using linear regression model, and multiple criteria were applied for candidates screening, including *P value*, *r*^2^, association directions, and etc. Expression information of the candidates for breast cancer was retrieved from a large patient cohort from The European Genome-phenome Archive database (EGAS00000000083) [53], and was also conducted to association analyses with *GATA3* expression with linear regression model as well.

All the *GATA3*-related genes were imported into the STRING, IntAct and BioGRID for protein-protein interaction network construction [54], and DAVID for pathway analyses [55].

### Plasmids of shRNA cloning, lentivirus production and stable cells constructions

Pairs of shRNA oligonucleotides for *GATA3* were annealed and ligated into the pLKO-TRC vector with AgeI and EcoRI digested and gel-purified. The constructed plasmids were verified by Sanger sequencing. Sequence information of shRNAs against our interested candidates were obtained from online information (http://www.sigmaaldrich.com/, Supplementary Table 7). Lentivirus was prepared with calcium phosphate-mediated transfection of 293T cells, which were cultured with 10% FBS contained DMEM medium. Lentivitral vectors were cotransfected with the helper vectors pCAGkGP1R, pCAG4-RTR2 and pCAG-VSV-G, and lentiviruses were purified by 0.45 um syringe filters. 697 cells were seeded into 6-well plates at a density of 1–2 million and infected with purified lentivirus particles. Polybrene (3 ul of 5 mg/ml stock solution) was added to the cells, followed by 3 ml of lentivirus solution. Cells were spin infected in 6-well plates for 1 h at 2000 rpm at 30°C. After cells and lentivirus co-incubated for 18 h at 37°C, the supernatant was removed by centrifugation and aspiration. Next, cells were resuspended in fresh 10% FBS contained RPMI medium, and incubated at 37°C for 72 h. Next, the knockdown stable cells were selected from infected cells with appropriate puromycin concentrations.

### RNA isolation and real-time PCR

RNA extractions for stable cells were performed with Animal Total RNA Isolation Kit (Foregene, RE-03013) according to the manual protocol and reverse transcribed into cDNA with PrimeScript^™^ RT reagent Kit with gDNA Eraser (TAKARA, RR047A). Real-time PCR was performed with PowerUp™ SYBR^®^ Green Master Mix (Applied Biosystems^™^, A25776) to estimate the knockdown efficacy of shRNA as well as the selected gene expression, and primer sequence information is listed in Supplementary Table 8.

## SUPPLEMENTARY FIGURES AND TABLES





## Abbreviations

ALL: Acute lymphoblastic leukemia
TCGA: The Cancer Genome Atlas
GWAS: genome-wide association studies
LCL: Lymphoblastoid cell line
AML: acute myeloid leukemia
CLL: chronic lymphoblastic leukemia
CML: chronic myeloid leukemia
GEO: Gene Expression Omnibus
