# Supplementary material for: Regulatory network of GATA3 in pediatric acute lymphoblastic leukemia
Source: Oncotarget. 2017 Mar 21;8(22):36040–53. doi: 10.18632/oncotarget.16424 (PMC5482637; doi:10.18632/oncotarget.16424)
Supplement: Supplementary file 1 [file oncotarget-08-36040-s001.pdf]

Regulatory network of *GATA3* in pediatric acute lymphoblastic leukemia

Supplementary Materials

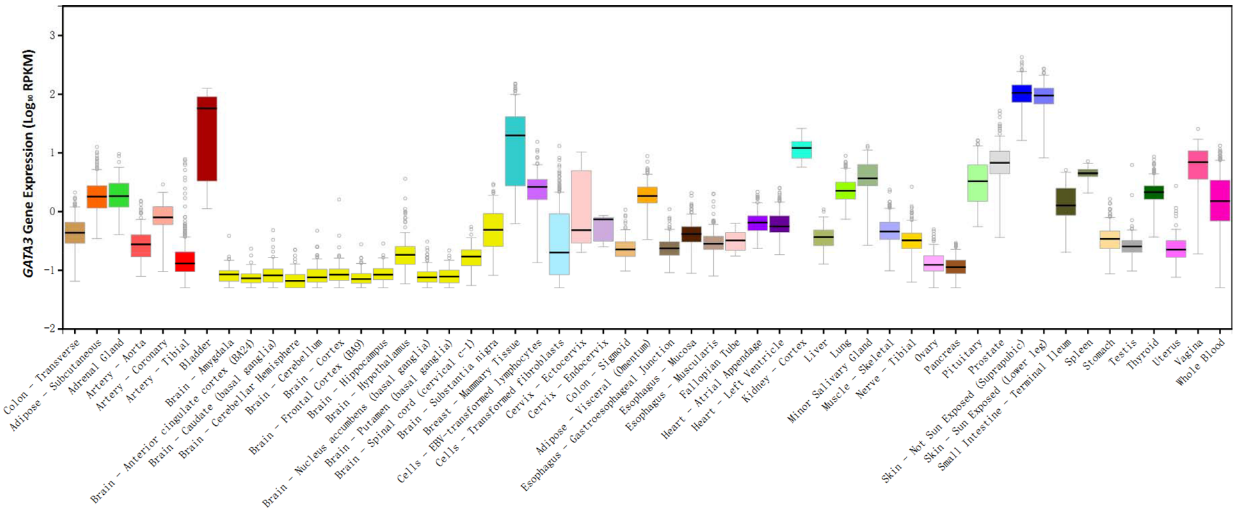

Supplementary Figure 1: Varied expression level of *GATA3* in different tissues.

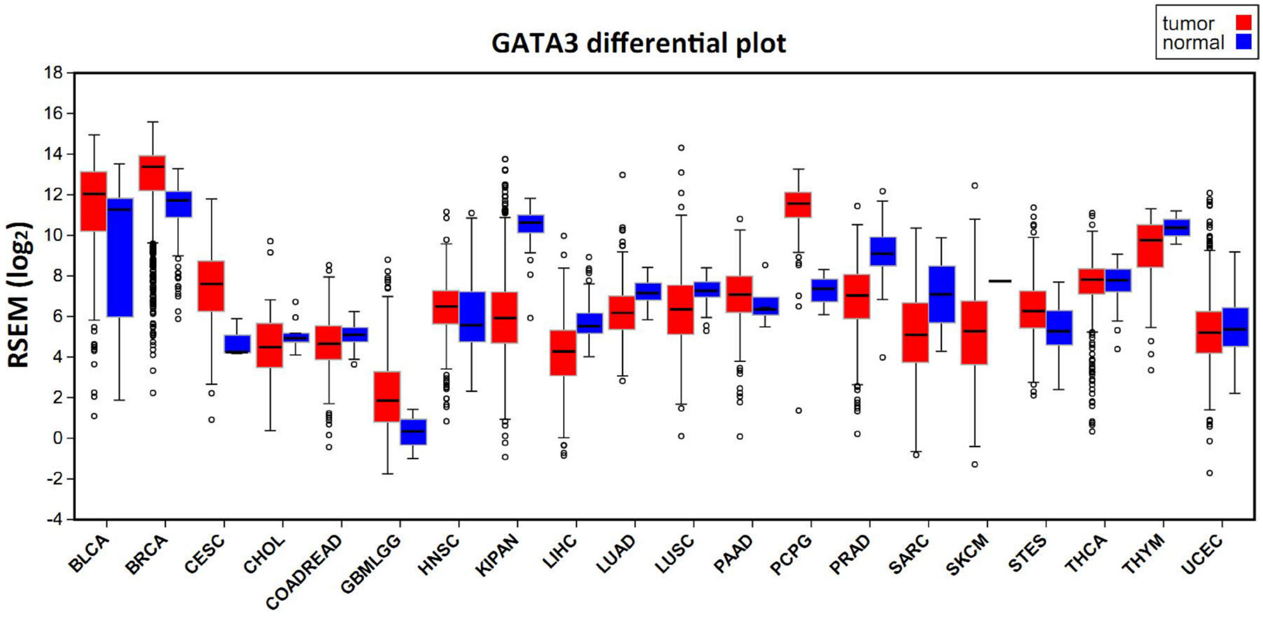

Supplementary Figure 2: *GATA3* expression in different cancer types from TCGA datasets, downloaded from http://firebrowse.org/. Significant differences (P = 0.05) between tumor and control were only seen in PCPG (pheochromocytoma and paraganglioma) and KIPAN (pan-kidney carcinomas) with opposite direction.

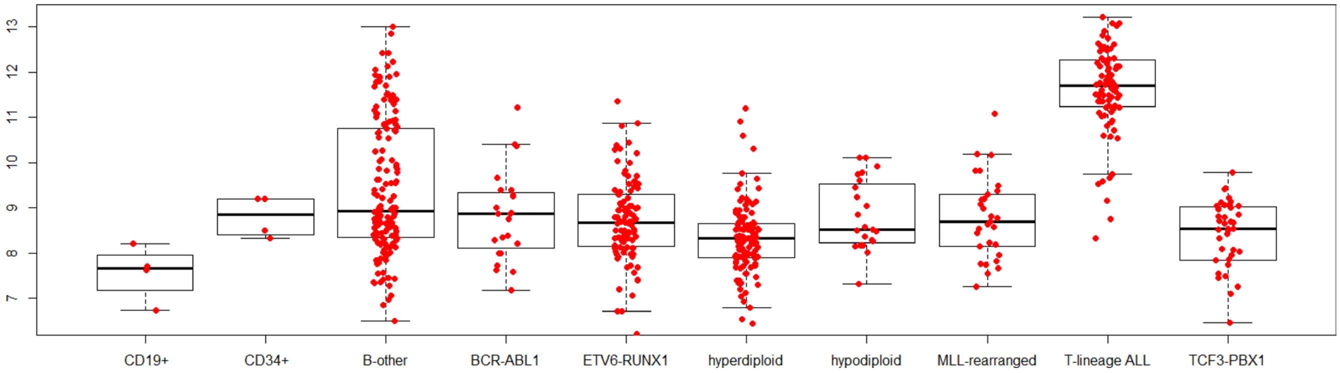

Supplementary Figure 3: *GATA3* expression in $CD19^+$ or $CD34^+$ cells from healthy individuals, T-ALL and different subtypes of B-ALL. ANOVA, $P < 0.05$.

Supplementary Table 1: Candidates of *GATA3* related genes in B-ALL. See Supplementary_Table_1

Supplementary Table 2: Functional clustering of *GATA3*-related genes

Category	Term	GeneCount	*P* value	Bonferroni *P* value	Genes list
Supplementary Table 2: Functional clustering of GATA3-related genes	Cyclin	4	0.002006438	0.09	CCND3, CCNH, CCNJL, CCNJ
GOTERM_MF_DIRECT	RNA polymerase II regulatory region sequence-specific DNA binding	11	1.84E-06	5.47E-04	ETV6, GATA3, RORA, SATB1, SOX13, ATF3, JARID2, MEF2C, NFIL3, STAT4, ZBED1

Supplementary Table 3: Candidates of GATA3 related genes in different subtypes (GSE33315). See Supplementary_Table_3

Supplementary Table 4: Candidates of GATA3 related genes in different types of leukemia and non-leukemic healthy bone marrow (GSE13204). See Supplementary_Table_4

Supplementary Table 5: Candidates of GATA3 related genes in breast cancer. See Supplementary_Table_5

Supplementary Table 6: Expression changes of the candidates in GATA3 overexpression cells

Gene	*P* value	EV-1	EV-2	GATA3-OE-1	GATA3-OE-2	
SAMSN1	0.009630153	4.331255	4.955194	7.787131	7.820278	+
ITGA6	0.002721363	8.067904	8.162796	9.611972	9.745022	+
ZNF44	0.02852521	6.107781	5.604447	7.296218	7.422691	+
ATP9A	0.014637422	5.704957	6.004722	7.072393	7.124633	+
IL9R	0.017284126	6.873853	7.048882	7.817419	7.999066	+
CCNJL	0.015008547	7.449637	7.652742	8.386895	8.461376	+
ETV6	0.001206087	9.194973	9.182852	10.01168	10.06964	+
ITM2A	0.005616151	6.966547	7.075683	7.860684	7.805649	+
MAST4	0.000699664	6.716645	6.682201	7.418738	7.403486	+
GATA3	0.020108823	7.736215	7.545323	8.303967	8.360753	+
AKAP13	0.00215624	8.295256	8.251765	8.927106	8.972565	+
GNA15	0.030525861	10.0625	10.03057	10.54918	10.76526	+
CBLB	0.000814048	8.171267	8.202226	8.779455	8.794131	+
PPP3CC	0.002340832	8.390239	8.443171	9.008248	8.988783	+
KIAA0182	0.005178245	8.213148	8.234017	8.770061	8.699232	+
HIPK1	0.00547994	10.15409	10.17168	10.62894	10.70152	+
MBOAT7	0.001470632	8.664957	8.645461	9.141216	9.110776	+
ATP2C1	0.031162598	7.829495	7.961018	8.301301	8.400515	+
SATB1	0.052773271	7.9569	8.327275	8.476987	8.663824	+
JARID2	0.006511339	9.249466	9.220243	9.628513	9.690912	+
SPTBN1	0.00922248	11.54285	11.59837	11.9137	11.95726	+
SOX13	0.063636772	6.493945	6.501388	6.721893	6.883419	+
MSL2	0.017829502	11.0028	10.97885	11.1876	11.24352	+
CHD4	0.102211029	10.17869	10.26785	10.34683	10.40206	+
FNDC3A	0.191862058	9.058537	8.992129	9.085381	9.161942	+
LEPROTL1	0.018155355	9.761321	9.746174	9.826989	9.843266	+
CCNH	0.080286925	8.337465	8.305736	8.418486	8.382834	+
PTP4A2	0.321089555	11.00637	11.01043	11.02212	11.11091	+
IGF1R	0.681022141	9.052861	8.918356	9.003118	9.033759	+
MME	0.184417312	12.26735	12.24425	12.19819	12.23192	−
PTK2B	0.71029827	8.776981	8.964705	8.878619	8.770291	−
PSD3	0.583029321	7.171178	7.051664	7.111632	7.00901	−
TRIM38	0.395546941	8.852402	8.998235	8.838618	8.854598	−
ZBED1	0.126959266	7.876415	7.801803	7.738536	7.748944	−
CEP68	0.11929844	7.458556	7.44757	7.317334	7.391541	−
MEF2C	0.296736136	10.2895	10.40803	10.19606	10.28994	−
IGFBP4	0.074011415	7.892481	7.96486	7.713647	7.786787	−
GABBR1	0.039327472	7.758965	7.83726	7.608704	7.603661	−
PRDX1	0.021824923	12.38752	12.38173	12.19995	12.13497	−
PTK2	0.043066598	8.607107	8.594789	8.259859	8.379831	−
SMTN	0.042409378	7.501192	7.638014	7.199994	7.25172	−
LAT2	0.020157378	9.610168	9.700043	9.229549	9.297812	−
ECM1	0.126372207	6.823502	6.775235	6.179746	6.527516	−

Supplementary Table 7: shRNA sequence against GATA3 and its related candidates

shRNA primer	shRNA sequence	Code
GATA3-shRNA-F1	AATTAGCCTAAACGCGATGGATATACTCGAGTATATCCATCGCGTTTAGGCTTTTTG	TRCN0000273991
GATA3-shRNA-R1	CCGGCAAAAAAGCCTAAACGCGATGGATATACTCGAGTATATCCATCGCGTTTAGGCT	
GATA3-shRNA-F2	AATTGTGGGCTCTACTACAAGCTTCCTCGAGGAAGCTTGTAGTAGAGCCCACTTTTTG	TRCN0000285105
GATA3-shRNA-R2	CCGGCAAAAAGTGGGCTCTACTACAAGCTTCCTCGAGGAAGCTTGTAGTAGAGCCCAC	

Supplementary Table 8: primers for real-time PCR detection

Gene	qPCR-primer	
ETV6	ETV6-qPCR-F	TGAGAAAATGTCCAGAGCCC
	ETV6-qPCR-R	ACTCTAGGTGCTCCAGACG
GATA3	GATA3-qPCR-F	GCGGGCTCTATCACAAAATG
	GATA3-qPCR-R	TCCCCATTGGCATTCCTC
ITM2A	ITM2A-qPCR-F	GAGGCTGACATTCGTGAGG
	ITM2A-qPCR-R	CATCAGATAGCAGTTCCCCAG
ITGA6	ITGA6-qPCR-F	CCTCCCTGAGCACATATTCG
	ITGA6-qPCR-R	CACCTCCAACTTCTCCATCTC
CBLB	CBLB-qPCR-F	GACGCCATGATTTGCCTTTAG
	CBLB-qPCR-R	ACGGACCAGTACACTTTATGC
TCL1A	TCL1A-qPCR-F	TTTCTGGCGCTTAGTGTACC
	TCL1A-qPCR-R	AACATCCCCATTGTAGGCTG
WT1	WT1-qPCR-F	TGTCAGCGAAAGTTCTCCC
	WT1-qPCR-R	GTTGTGATGGCGGACTAATTC
GAPDH	GAPDH-qPCR-F	AGCCACATCGCTCAGACAC
	GAPDH-qPCR-R	GCCCAATACGACCAAATCC
